# Lumbar Facet Joint Arthritis Is Associated with More Coronal Orientation of the Facet Joints at the Upper Lumbar Spine

**DOI:** 10.1155/2013/693971

**Published:** 2013-10-23

**Authors:** Thorsten Jentzsch, James Geiger, Stefan M. Zimmermann, Ksenija Slankamenac, Thi Dan Linh Nguyen-Kim, Clément M. L. Werner

**Affiliations:** ^1^Division of Trauma Surgery, Department of Surgery, University Hospital Zürich, Rämistrasse 100, 8091 Zürich, Switzerland; ^2^Institute of Diagnostic and Interventional Radiology, University Hospital Zürich, Rämistrasse 100, 8091 Zürich, Switzerland

## Abstract

We retrospectively analyzed CT scans of 620 individuals, who presented to our traumatology department between 2008 and 2010. Facet joint (FJ) arthritis was present in 308 (49.7%) individuals with a mean grade of 1. It was seen in 27% of individuals ≤40 years and in 75% of individuals ≥41 years (*P* < 0.0001) as well as in 52% of females and 49% of males (*P* = 0.61). Mean FJ orientation was 30.4° at L2/3, 38.7° at L3/4, 47° at L4/5, and 47.3° at L5/S1. FJ arthritis was significantly associated with more coronal (increased degree) FJ orientation at L2/3 (*P* = 0.03) with a cutoff point at ≥32°. FJs were more coronally oriented (48.8°) in individuals ≤40 years and more sagittally oriented (45.6°) in individuals ≥41 years at L5/S1 (*P* = 0.01). Mean FJ asymmetry was 4.89° at L2/3, 6.01° at L3/4, 6.67° at L4/5, and 7.27° at L5/S1, without a significant difference for FJ arthritis. FJ arthritis is common, increases with age, and affects both genders equally. More coronally oriented FJs (≥32°) in the upper lumbar spine may be an individual risk factor for development of FJ arthritis.

## 1. Introduction

A functional spinal unit consists of anteriorly located adjacent vertebrae separated by an intervertebral disc and posteriorly located facet (zygapophyseal) joints (FJ) [1]. FJs are composed of an inferior articular process, facing anteriorly, and a superior articular process, facing posteriorly, of two adjacent vertebrae [2]. Being synovial-lined, diarthrodial, and freely moveable functional units, they transmit shear forces and help the intervertebral discs in carrying about 16% of the vertical load [3, 4]. FJ orientation planes differ at various levels, with a more sagittal and curved orientation for resistance against axial rotation in the upper compared to a more coronal and flat orientation for resistance against flexion and shearing forces in the lower lumbar segments [5, 6]. FJ asymmetry or tropism describes the asymmetry of the left and right FJ angle [7, 8]. 

Low back pain is one of the most common health problems [9]. It affects up to 85% of people at least once during their lifetime and up to 5% chronically [10]. Even though etiologies of low back pain are multifactorial [11], FJ arthritis is common and affects at least 50% of the population [12]. After Ghormley [13] first described a facet syndrome in 1933, there has been an ongoing debate [14, 15] about the possible association low back pain and FJ pathology [16]. FJs are synovial covered joints with hyaline cartilage [17] and innervated by the medial branches of the dorsal rami from two levels [18, 19]. Recently, it has been shown that inflammatory chemical mediators are increased in degenerated FJs [20]. In order to investigate the association of low back pain and FJ pathology, most studies [21–25] successfully utilized FJ (nerve) blocks and its associated pain relief. Thus, there is convincing evidence that FJ pain plays an important role in low back pain [26, 27] and occurs in up to 45% of individuals [25]. 

However, controversies still exist in the following issues. In general, study samples have been rather small for FJ arthritis on CT scans, which is especially true for the prevalence of FJ arthritis, particularly in younger individuals [28–36]. Gender predilection has not been reported consistently [12, 15, 29, 37]. It also remains unclear whether FJ arthritis is associated with FJ orientation and/or FJ asymmetry, and if so, at which level [1, 6–8, 32, 38–48]. Previous studies [32, 49] have only reported an increase in FJ arthritis with more sagittally oriented FJs at the lower lumbar spine. Yet, it is unknown if changes in FJ orientation at the upper lumbar spine lead to FJ orientation at the lumbar spine. Therefore, our goal was to clarify these remaining issues by quantifying the degree of radiographically detectable (1) FJ arthritis on CT scans of the lumbar spine from L2-S1 in regards to (2) age, (3) gender, (4) FJ orientation, and (5) FJ asymmetry.

## 2. Materials and Methods

The study has been approved by the institutional review board (ethical committee no. KEK-ZH-Nr.2011-0507). We retrospectively analyzed CT scans of 620 individuals (2480 functional units), with a mean age of 42.5 (range, 14–94) years, who presented to our traumatology department and underwent a whole body CT scan, including the pelvis and lumbar spine, between 2008 and 2010. A dual-source computed tomography scanner (Somatom Definition, Siemens Healthcare, Forchheim, Germany) was used [50]. Our study utilized CT scans instead of plain radiographs or magnetic resonance imaging, because they are more accurate in displaying FJs on axial planes [51, 52]. FJs of the lumbar spine were evaluated between the second lumbar and the first sacral level [53]. Axial planes with the largest intersecting set of the superior and inferior FJ process were chosen. 

**Figure 1:**
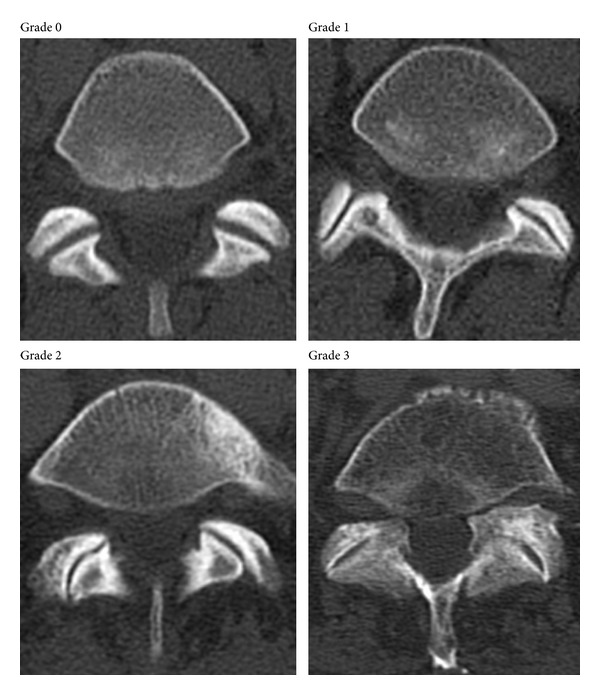
Grading scale for FJ arthritis. Grade 0 = normal FJ. Grade 1 (mild) = joint space narrowing, grade 2 (moderate) = sclerosis, and grade 3 (severe) = osteophytes.

**Figure 2:**
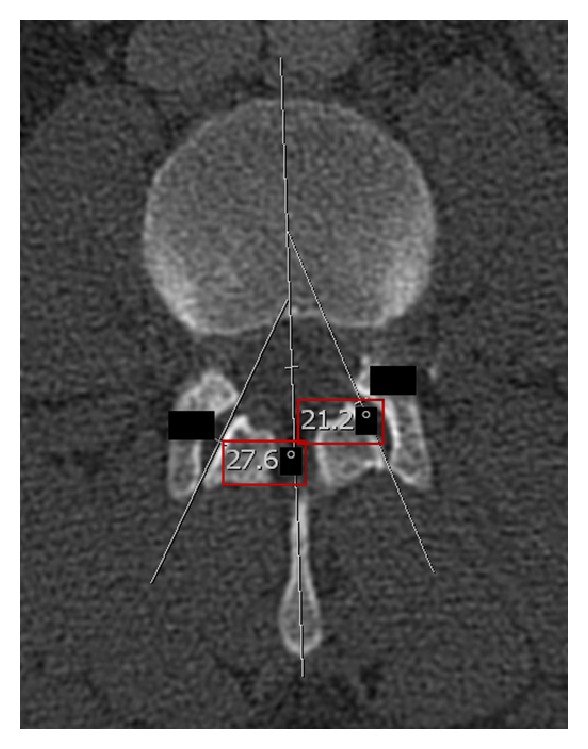
Measuring technique for FJ orientation. FJ orientation in the axial plane was evaluated by measuring the angle between the midline of the sagittal plane and the midline of the FJ. Coronal FJ orientation is shown on the left side, whereas sagittal orientation including measurement of FJ orientation is shown on the right side. The red box indicates the value for FJ orientation. The blacked out numbers were disregarded because they were created automatically by our software and contained irrelevant information.

(1) Assessment of FJ arthritis was carried out as previously described in similar studies, where a grading scale described by Pathria [29, 54] was used. Grade 0 (normal) indicates a normal facet joint, whereas grades 1–3 display increasing signs of FJ arthritis with each grade including signs of the lower grade. Grade 1 (mild) shows joint space narrowing, grade 2 (moderate) demonstrates sclerosis, and grade 3 (severe) reveals osteophytes [55] (Figure 1). (2) Individuals were grouped into those ≤40 and ≥41 years. (3) Gender was also evaluated. (4) FJ orientation in the axial plane was evaluated by measuring the angle between the midline of the sagittal plane and the midline of the FJ as described by Schuller et al. [56, 57] (Figure 2). FJ orientation (Figure 2) was determined on axial CT planes of the lumbar spine using the AGFA Impax viewer. The midline of the sagittal planes corresponds to a line drawn through the center of the vertebral body and spinous process. Therefore, each FJ was compared against this line. The midline of FJs was evaluated on axial cross-sections where the largest part of the joint, that is, most parts of the superior and inferior articular facets were visible. The overall FJ orientation was calculated by averaging the angles between the right and left side of the FJs. We used absolute angles, indicating that we did not consider rotation in one direction as positive and rotation in the opposite direction as negative. The FJ orientation was labeled as coronal if angles were >45°, sagittal if angles were ≤45°, and anisotropic if one side was over and the other side under 45° [58]. (5) FJ asymmetry was determined as the absolute difference between the right and left FJ angle and categorized into four groups determined according to their 50th, 75th, and 95th percentile, for example, group one includes 50% of the sample, group two 25%, group three 20%, and group four 5%.

All statistical analyses were performed by the Institute for Social and Preventive Medicine, Division of Biostatistics at the University of Zürich, using the R program [59]. Several different statistical approaches were applied to test the null hypothesis [60]. This study is an observational study, which means that analysis follows a descriptive and exploratory form. Therefore, *P* values are interpreted as a quantitative measure of the evidence against the null hypothesis. As a rough guideline, we assumed weak evidence against the null hypothesis for *P*-values ≥0.01 and <0.1, modest evidence against the null hypothesis for *P* values between ≥0.001 and <0.01, and strong evidence against the null hypothesis for *P* values <0.001. Therefore, correction for multiple comparisons has been assessed. The *G*_2_ test was used for the following models: FJ arthritis, versus (2) age (categorized), (3) gender and (5) FJ asymmetry. The *G*_2_-test was used to test the association between ordinal outcomes and nominal explanatory variables. Besides the usual properties of a statistical test, the *G*_2_ test also provides a decomposition of the total test value *G*_2_ into the ordinal levels of the outcome variable, and can therefore be used to determine the threshold of the ordinal levels. For example, the decomposition of the *G*_2_-value for the 4 degrees of FJ arthritis, which is an ordinal measure, is as follows: *G*_2_ = *G*_2_0;  1 + *G*_2_01;  2 + *G*_2_012;  3, which means that the total *G*_2_-value can be written as *G*_2_-value of a comparison between FJ arthritis 0 and 1, plus a *G*_2_-value of a comparison between FJ arthritis 0 + 1 and 2, plus a *G*_2_-value of a comparison between FJ arthritis 0 + 1 + 2 and 3. If the equation would be 100 = 10 + 30 + 100 for a certain explanatory variable, the largest difference occurs between 30 and 100. Therefore, patients with a degree of 3 in regard to FJ arthritis show the largest difference with respect to this explanatory variable. A *χ* ^2^-test was applied to test the association between a nominal outcome and a nominal explanatory variable. The *χ* ^2^-test was used for the following models: (4) FJ orientation (categorized) versus age (categorized) and gender, as well as (5) FJ asymmetry versus age (categorized) and gender. The proportional odds model was used for (1) FJ arthritis versus (4) FJ orientation. We also calculated the cut-off point for FJ arthritis by using the ROC curves analysis. Afterwards we performed a univariate as well as a multivariate logistic regression analysis by grouping the patient population according to the cut-off point. Age and gender were defined as potential confounder for the multivariate regression analysis. 

## 3. Results

*(1) Arthritis*. Of our 620 individuals, who were evaluated for radiological FJ arthritis on axial planes of CT scans from L2-S1, 308 (49.7%) individuals showed signs of FJ arthritis. The mean grade of FJ arthritis was 1.310 (50.0%); individuals were not affected by FJ arthritis (grade 0), 103 (16.6%) individuals presented with grade 1, 107 (17.3%) individuals with grade 2, and 98 (15.8%) individuals with grade 3 (Table 1). Two (0.3%) individuals could not be evaluated for FJ arthritis because spondylodesis had been performed or appropriate planes had not been reconstructed adequately.

**Figure 3:**
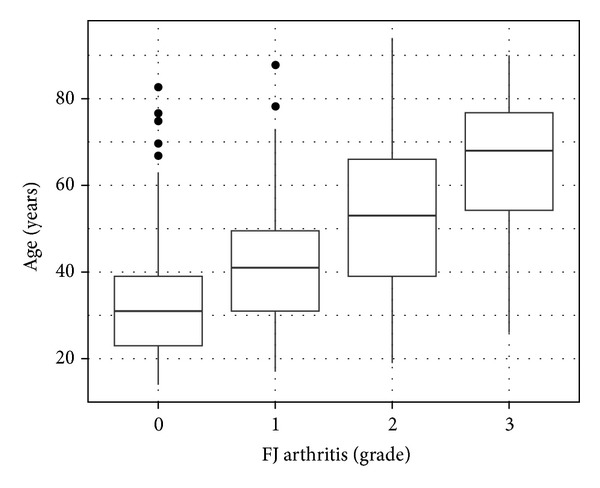
Grade of FJ arthritis and age. This figure describes the increasing grade of FJ arthritis with age.

*(2) Age*. Separated into two age groups, our study included 330 (53.2%) individuals ≤40 years and 290 (46.8%) individuals >40 years. FJ arthritis significantly differed between age groups, with elderly individuals being more commonly affected (*P* < 0.0001) (Figure 3). All 4 degrees of FJ arthritis were found in both age groups (≤40 years, >40 years) but with different proportions. FJ arthritis was present in 27% of individuals in the age group ≤40 years. In contrast, FJ arthritis was found 75% of individuals in the age group >40 years. Furthermore, FJ arthritis manifested in 95% of individuals in the age group ≥65 years, which included 97 individuals. The *G*_2_0,12; 3-value indicates that comparison of the first 3 groups of FJ arthritis with a degree of 0, 1, and 2 to the most severe group of FJA with a degree of 3 showed the largest gap in age (207 = 30 + 58 + 119). This suggests that severe FJ arthritis seemed to be more likely in elderly individuals.

*(3) Gender*. There were 202 females (32.6%) and 418 males (67.4%). FJ arthritis did not show significant gender predilection, even if separated into age groups. 52% of females and 49% of males displayed signs of FJ arthritis (*P* = 0.61). Females presented with a mean FJ arthritis of 1.07, compared to 0.95 in males. Each grade of FJ arthritis included a similar number of females and males. Grade 0 affected 48% of females and 51% of males, grade 1 affected 15% of females and 17% of males, grade 2 affected 19% of females and 17% of males, and grade 3 affected 18% of females and 15% of males.

**Figure 4:**
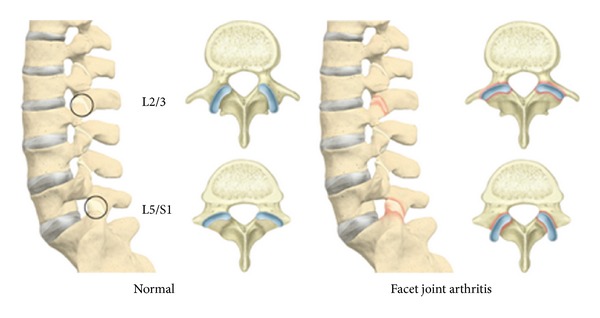
FJ arthritis and FJ orientation. On the left side, sagittally oriented FJs at L2/3 and coronally oriented FJ at L5/S1 are associated with normal FJs at the lumbar spine. The right side illustrates inversely oriented FJs with arthritic FJs at the lumbar spine, namely, coronals oriented FJs at L2/3 and sagittally oriented FJs at L5/S1.

**Figure 5:**
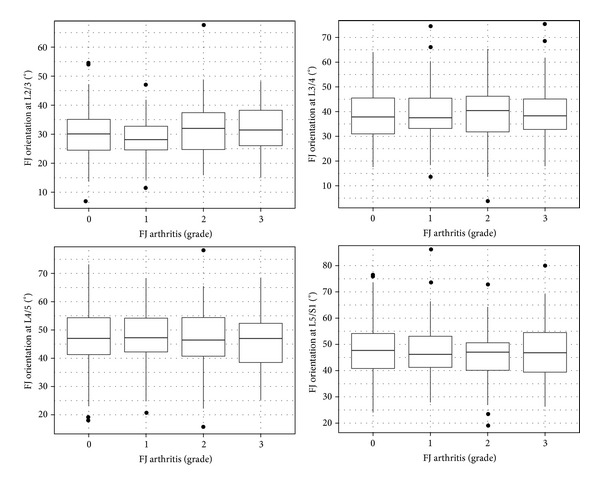
Cephalocaudal change in FJ orientation. There was a steady progress from a sagittal toward a more coronal FJ orientation at the lumbar spine in a cephalocaudal fashion, namely, 30° at L2/3 and 47° at L5/S1.

*(4) Orientation*. Mean FJ orientation was measured as 30.4° (SD 7.7°, range 7.4–66°) at L2/3, 38.7° (SD 9.6°, range 4.5–73.7°) at L3/4.47° (SD 9.8°, range 16.2–76.4°) at L4/5 and 47.3° (SD 9.9°, range 19.6–84.4°) at L5/S1. FJs of the proximal lumbar levels were more sagittally oriented compared to those at distal lumbar levels, which were more coronally oriented (Figures 4 and 5). Thus, there was a cephalocaudal trend of an increasing degree of FJ orientation. 

FJ arthritis was significantly associated with more coronal, that is, increased degree of FJ orientation at L2/3 (mean FJ orientation of 30.1° without FJ arthritis (grade 0) versus mean FJ orientation of 32.1° with FJ arthritis (grade 3) (*P* = 0.03, OR 1.021 (95%-CI 1.002–1.014))) (Figure 4). The cut-off point was ≥32°. This means that more coronally oriented FJs, that is, ≥32°, at this level were associated with a higher radiological degree of FJ arthritis. No significant association between FJ arthritis and FJ orientation could be established at the other levels. There was a significant difference for FJ orientation in our age groups at L5/S1 (*P* = 0.01), where more coronal FJ orientation (48.8°) manifested in individuals ≤40 years and a more sagittal FJ orientation (45.6°) was present in individuals >40 years. No significant difference was found in FJ orientation and age groups at other levels (30.0° versus 31.00° (*P* = 0.61) for L2/3, 43.6° versus 42.1° (*P* = 0.41) for L3/4 and 48.1° versus 45.9° (*P* = 0.13) for L4/5). There were no significant differences for FJ orientation and gender (*P* = 0.13–0.73).

*(5) Asymmetry*. The mean values for FJ asymmetry were calculated as 4.89° at L2/3, 6.01° at L3/4, 6.67° at L4/5, and 7.27° at L5/S1. There was no difference between FJ arthritis and FJ asymmetry (*P* values = 0.11 for L5/S1, 0.26 for L4/5, 0.10 for L3/4 and 0.17 for L2/3). There were no significant differences in age groups for each level (*P* = 0.35 at L2/3, 0.23 at L3/4, 0.27 at L4/5, 0.28 at L5/S1). However, there was modest evidence that FJ asymmetry is more common in females than in males at L5/S1 (*P* = 0.01) but not at the other levels (*P* = 0.47, 0.91 and 0.33 for L2/3, L3/4 and L5/S1). FJ asymmetry also increased in a craniocaudal fashion.

## 4. Discussion

Our study investigated one of the largest samples of CT scans with regard to FJ arthritis in the literature. As hypothesized we were able to show that (1) radiological appearance of FJ arthritis is a very common entity, affecting nearly half of all individuals, (2) increases with age, (3) does not display gender predilection, (4) was significantly associated with coronal, that is, increased degree of FJ orientation at L2/3, and (5) is not correlated with FJ asymmetry.

Limitations of our study attribute to the fact that all individuals presented to a trauma department. Even though a selection bias may be assumed, we did not include individuals with a fracture of the lumbar spine. We were not able to check for intra- or interrater reliability, but measurements were carried out by two trained specialists in this field. Furthermore, the measuring technique has been described before and did not require validation. We did not pay special attention to degenerative disc disease since this has been investigated in previous studies [7, 34, 49]. Another problem is caused by the parallax effect. It has been advocated [61, 62] that the spinous process may be an unreliable anatomic midline marker because anatomic variations, such as scoliosis, in the relationship between the anterior vertebral body and posterior spinous process may skew the interpreter's view on X-rays. However, this parallax effect is much smaller for more accurate images of CT scans, which we used for our evaluation. The measuring technique used in our study has been well established in previous studies [6, 32, 38, 42]. Anyhow, this issue does not affect our evaluation of FJ orientation because we calculated the mean of both sides and did not interprete each side independently. We do acknowledge that setting of a wrong midline may pose a problem in regard to FJ tropism, but so far no solution to this issue has been presented in the literature. The interesting finding that the interpedicular midpoint is the most accurate guide to the coronal midline by Mistry and Robertson. [62] could be implemented in future studies. Due to the retrospective nature of this study, we were not able to investigate which individuals showed clinical signs of FJ arthritis. Anyway, even though there is an ongoing debate [14–16, 21–25] whether radiologic proof of FJ arthritis is clearly associated with back pain, it was not the purpose of our study. Due to the cross-sectional design, we were unable to determine whether more coronally oriented FJs at L2/3 lead to FJ arthritis or the other way around. However, we believe that these changes in FJ orientation go along with FJ arthritis rather than being a manifestation of aging, because we did not find a significant association between changes in FJ orientation at L2/3, and age, or a combined significant association at the other levels. This interesting topic may be evaluated in future longitudinal studies. We did not specify the exact level or side of FJ arthritis since all levels and sides seemed to be affected in a similar fashion, with lower levels being slightly more frequently affected [12]. Even though our study included a similar number of individuals under and over 40 years, it comprised nearly twice as many males, which may be attributed to the fact that males are injured more often and are overrepresented in a trauma population [60].

*(1) Arthritis.* FJ arthritis was found in almost 50% of individuals in our study. This is similar to previous studies. Eubanks et al. [12] studied 647 cadavers and reported the following prevalence of FJ arthritis: 53% at L1/2, 66% at L2/3, 72% at L3/4, 79% at L4/5, and 59% at L5/S1. Kalichman et al. [28] also reported a high prevalence of FJ arthritis, namely, 64.5%, in a study 187 individuals from the 3,529 participants enrolled in the Framingham Heart Study who were assessed for aortic calcification with CT scans. Looking at 361 patients, Suri et al. [29], found an even higher prevalence of FJ arthritis, where 22% presented with isolated posterior (FJ) arthritis and 57% showed signs of posterior and anterior arthritis. Our results support the fact that FJ arthritis is a common pathology of the spine. The individuals in our study displayed a lower mean age of 42.5 years compared to the mean age of 52.6 years and 58.0 years in previously mentioned studies by Kalichman et al. [28] and Suri et al. [29] respectively, whereby Eubanks et al. [12] did not report a mean age. This explains why our results for FJ arthritis affect a smaller number of individuals.

*(2) Age*. We showed that FJ arthritis was present in 27% of individuals ≤40 years. Our results also illustrated a significant association of FJ arthritis and increasing age (Figure 3). 75% of our individuals ≥41 years and 95% ≥65 years presented with FJ arthritis. Likewise, previous studies [30, 31, 33, 35, 36] have revealed that FJ arthritis arises at a young age and is found in more than 50% of individuals over 40 years. In a study by Swanepoel et al. [34], who investigated individuals under 30 years, macroscopic cartilage fibrillation was more pronounced in FJs than in other joints, such as hip, knee, and ankle. In Eubanks' et al. study [12], FJ arthritis was present in 57% of individuals between 20–29 years, 82% between 30–39-years, 93% between 40–49 years, 97% between 50–59 years, and 100% over 60 years. In an ancillary to the Framingham study, Suri et al. [29] investigated 361 individuals and reported a correlation of FJ arthritis with age (OR 1.09). 89% of individuals over 65 years suffered from FJ arthritis. In a different study of 57 cadaveric specimens of spinal-disease-free organ donors, Li et al. [33] stated that FJ arthritis increased with age and no spine was completely spared by FJ arthritis over the age 42 years. These results all report the same fact and are not surprising since FJs transmit shear forces, carry about 16% of the vertical load, and tend to be subject to wear and tear [3, 4].

*(3) Gender*. Our study did not find a significant association of FJ arthritis and gender, even though females were slightly more commonly affected (52%) than males (49%). Similarly, in a study by Abbas et al. [37], FJ arthritis did not show gender predilection in 215 individuals, which was investigated from L3-S1 on CT scans. In a study of 188 individuals by Kalichman et al. [15], females were slightly more commonly affected by FJ arthritis than men, namely, 67% versus 60%. However, this difference was not significant. According to Suri et al. [29] females (OR 1.86) were more commonly affected, too. On the other hand, men had a higher prevalence of FJ arthritis in a cadaveric study of 645 spines (*P* < 0.001) by Eubanks et al. [12], but unfortunately, no percentages were stated. Overall, there is more evidence that gender cannot be counted on as a risk factor for FJ arthritis. This is surprising, because most of the males, especially those presenting to our traumatology department, are working in hard labor jobs. Being synovial-lined, diarthrodial, and freely moveable functional units, they transmit shear forces and help the intervertebral discs in carrying about 16% of the vertical load. Therefore, most of the weight is carried by the intervertebral disc. Even though hard labor may mainly affect arthritis of the intervertebral disc, a recent study has shown that estrogen also leads to arthritis of the intervertebral disc [63]. In conclusion, these two factors may balance each other out. Other potential factors that cause increased shear forces in women may play a role as well, such as scoliosis, weaker musculature, and carrying heavier weights in relation to their muscle strength, including pregnancy and carrying shopping bags [64].

*(4) Orientation*. Our results point out that increased FJ arthritis was significantly associated with a higher degree of FJ orientation, indicating a more coronal FJ orientation, at the upper lumbar spine, namely, L2/3 (*P* = 0.03). Interestingly, the cut-off point was ≥32°, indicating that more coronally oriented FJs, that is, ≥32° were associated with a higher radiological degree of FJ arthritis at this level. Even though not significant, the same trend was observed for L3/4 (OR 1.009), while our results were equivocal for the lower lumbar spine. Therefore, coronally oriented FJs at L2/3 may present a surrogate for FJ arthritis later on in life.

The correlation of FJ arthritis and FJ orientation has only been reported for the lower part of the lumbar spine. A significant association between FJ arthritis and sagittal FJ orientation of the lower lumbar spine was found in a study of CT scans with 188 individuals by Kalichman et al. [6] and a MRI study if 111 individuals by Fujiwara et al. [38]. Likewise, a recent CT study of 123 individuals by Liu et al. [32] linked FJ arthritis to more sagittally oriented FJs at L4/5 and L5/S1. Our novel finding of increased FJ arthritis with more coronally oriented FJs at the upper lumbar spine might be attributed to the specific function of FJs at different lumbar levels. Normal FJ orientation planes differ at various levels, with a more sagittal and curved orientation for resistance against axial rotation in the upper compared to a more coronal and flat orientation for resistance against flexion and shearing forces in the lower lumbar segments [5, 6]. If the upper lumbar segments display more coronally oriented FJs, they are more prone to FJ arthritis because they are not designed to withstand repeating axial rotation. Another theory by Dunlop et al. [65] hypothesizes that aging leads to increased stress in the anteromedial part of the FJ due to repetitive abrasion during flexion and rotation and therefore changes the morphology of FJs, resulting in increased sagittal orientation [42]. Importantly, inverse orientation of the normal state, namely, coronally oriented FJs at the upper and sagittaly oriented FJs at the lower lumbar spine may be independent risk factors for FJ arthritis (Figure 4).

In our study, more coronal FJ orientation was present in individuals ≤40 years, and a more sagittal FJ orientation manifested in individuals ≥41 years at L5/S1. This is in line to previous study by Wang and Yang [42], who noted that degenerative spondylolisthesis, which has been associated with sagittal FJ orientation in several reports [6, 40, 41], was accompanied by a negative correlation of age and coronally oriented FJs (*r* = −0.4555) through investigation of the orientation of FJs at L4/5 in 300 individuals at different age groups. Masharawi et al. [43] did not find an association between FJ orientation and age studying 240 human vertebral columns. These FJ changes may be attributed to degenerative wear and tear, either at the FJs or at the intervertebral disc, and resulting traumatic change FJ orientation into a more sagittal alignment [38]. Like previous studies by Wang and Yang [42] and Masharawi et al. [43], we also did not find an association between gender and FJ orientation. Besides, we were able to show a significant steady progress from a sagittal toward a more coronal FJ orientation in a cephalocaudal fashion, namely, 30° at L2/3 and 47° at L5/S1 (Figures 4 and 5). This is in line with an ancillary CT study of the Framingham Heart Study with 3529 individuals by Kalichman et al. [6], who also showed an increasing FJO from L3-S1.

*(5) Asymmetry*. Our values for FJ asymmetry are in line with previous studies [40], where FJ asymmetry was commonly under 7°. We were not able to show a correlation between FJ arthritis and FJ asymmetry. This is in line with most previous studies [6, 7, 40], such as by Boden et al. [7], who studied 140 individuals with CT scans. Likewise, Grogan et al. [40] studied 21 cadavers and a total of 104 FJs with CT scans and did not find an association between FJ arthritis and FJ asymmetry. On the other hand, a single paper by Kong et al. [46] stated that FJ arthritis was associated with FJ asymmetry at L4/5 but not at L3/4, and L5/S1 in an MRI study of 300 individuals. Moreover, there was no association of FJ asymmetry and age in our study. Previous studies [6, 46, 47] have yielded the same results. Overall, we could not find evidence for previous hypotheses, which attributed FJ asymmetry to asymmetric mechanical stress or inborn deformities. Our findings are supported by a study of dried vertebrae of 240 humans by Masharawi et al. [48], where FJ asymmetry was considered a normal characteristic of the thoracolumbar spine.

However, in our study, FJ asymmetry was significantly more common in females than in males at L5/S1, which is in contrast to the study by Kong et al. [46], who did not find a meaningful relationship. Interestingly, there was evidence that FJ asymmetry increases cephalocaudally, with a mean of 4.89° at L2/3 and 7.27° at L5/S1. This indicates that FJ asymmetry is less common in sagittally oriented and more common in coronally oriented FJs, namely, the lower lumbar levels. Accordingly, Cassidy et al. [8] and Masharawi et al. [44] stated that FJ asymmetry is more commonly found in coronally oriented FJs. This may be explained by the increased load and degenerative changes at the lower lumbar spine [66], which may lead to uncontrolled changes of the FJs. This may affect women more commonly due to changes in estrogen or other unknown factors [63].

## 5. Conclusion

In conclusion, FJ arthritis is common affecting about half of individuals, increases with age, and affects both genders equally. Coronally oriented facet joints (≥32°) in the upper lumbar spine, namely, at L2/3 may be an individual risk factor and surrogate for development of FJ arthritis in the entire lumbar spine, which is worth further investigations. Besides, coronal FJ orientation increases craniocaudally, while sagittal orientation at the lower lumbar spine increases with age. FJ asymmetry is not associated with FJ arthritis, is more common in females at the lower lumbar spine, and also increases in a craniocaudal fashion.

## 6. Disclosure

Each author certifies that he or a member of his or her immediate family has no funding or commercial associations that might pose a conflict of interest in connection with the submitted paper. Each author certifies that his institution approved the human protocol for this investigation and that all investigations were conducted in conformity with ethical principles of research.

## Figures and Tables

**Table 1:** Prevalence of facet joint (FJ) arthritis.

Grade	Patients (absolute number)	Patients (percentage)
0	310	50
1	103	16.6
2	107	17.3
3	98	15.8
